# Digital transformation of clinical trials with remote patient monitoring solutions

**DOI:** 10.1038/s44401-026-00161-5

**Published:** 2026-10-14

**Authors:** Elisabeth Piault, Ramya Palacholla, Simon Pulman-Jones, Emmett Alton Sartor, Sergio Simón, Jill Bell

**Affiliations:** 1 Evinova, Gaithersburg, MD USA; 2 AstraZeneca, Wilmington, DE USA; 3 AstraZeneca, Barcelona, Spain; 4 Evinova, Cambridge, UK; 5 Present Address: Emergence Now, Cambridge, UK

**Keywords:** Health care, Medical research

## Abstract

Remote patient monitoring (RPM) is becoming increasingly feasible to implement in clinical trials. A framework is presented for developing RPM digital solutions incorporating the needs and perspectives of patients and healthcare professionals, and regulatory requirements/guidance. This framework is illustrated with recent examples of RPM deployments in the context of skin toxicities and stomatitis. Strategies to overcome challenges in the adoption of RPM solutions in clinical trials are discussed.

## Introduction

In clinical trials following a site-centric model, participants are required to travel for assessments and to receive investigative or control treatment, which imparts a time and logistical burden on patients^1^. There are often challenges related to recruitment and retention in clinical trials, particularly with regard to long-term follow-up, that ultimately lead to underrepresentation of some demographic groups, creating geographic and socioeconomic disparities in trial participation^1^. An observational cohort study of 58 oncology trials showed that within 2 years of enrolment, approximately 9% of participants withdrew consent, with most of these withdrawing consent early in follow-up^2^. Additionally, the reliance on scheduled study site visits for retrospective adverse event reporting might result in inaccuracies and delays in symptom reporting and toxicity detection^3–5^. In recent years, there has been increased adoption and growing recognition of the potential benefits of decentralized or hybrid approaches in clinical trials^6–8^. Technologies such as consumer-friendly medical devices support electronic biomarker data collection with minimal or no clinical supervision; as such, these could help to make clinical trials more accessible and inclusive, and less burdensome^6^.

Over the past decade, remote patient monitoring (RPM) has involved the use of digital technologies for the collection of health-related data from patients, often outside the point of care, and for their transmission to healthcare professionals (HCPs) to inform their understanding of the patient’s health status^9^. RPM enables symptoms and certain signs to be reported between visits while the patient is at home, in real time, and without the need for hands-on involvement of physicians, pharmacists, and nurses^10^. RPM has been enabled by advances in small devices and digital technology, including sensor-based measurements using wearable devices, which can be non-invasive (e.g., pulse oximeters for oxygen saturation measurements)^11,12^ or invasive through home sampling or home testing (e.g., COVID-19 diagnosis)^13^. Mobile application (app)-based tools (e.g., weight management apps to track weight, food intake, and physical activity) have also advanced RPM development^14^.

Historically, patients with an established or suspected diabetes diagnosis typically took blood glucose measurements using manual capillary blood tests, logging measurements in notebooks and sharing data with HCPs during in-person visits or via telephone. Advancements in technology have enabled data collection in patients with diabetes to be tailored to their circumstances, to be either episodic or continuous, and to be collected via handheld, worn, or implanted sensors and transmitted to HCPs via smartphones or wearable, regulatory-approved medical devices connected to online data-sharing platforms^9^. These systems can also be closed-loop, with sensor-augmented insulin pumps to adjust insulin administration automatically in response to hyper-/hypo-glycaemia detected by the sensor. Compared with clinic visits, such technology reduces the need for in-person interactions with HCPs while increasing potential communication and monitoring, and it also enables patient self-monitoring and self-management, which increases patient autonomy and convenience and diminishes patient burden^15^. As part of the patient medical record, data collected using this technology also provides more granularity in the health status of patients to inform decision-making for treatment management^16,17^.

Many of the benefits of RPM in routine clinical care may be applicable to clinical trials, because providing insights into patients’ health status remotely while they are at home not only supports delivery of care by HCPs, but also provides more granular data that could support the totality of evidence of treatment benefit for market access. For example, real-time symptom tracking may facilitate the identification of suspected treatment toxicity events at an early stage, providing the opportunity for in-person follow-up for clinical evaluation or diagnosis, rather than relying on patients’ retrospective and potentially selective self-reporting during in-person or virtual visits. Remote monitoring could also support prophylactic protocols, helping patients to manage expected side effects and to stay engaged with the trial. In oncology, clinical trials involving an RPM intervention have demonstrated improvements in survival, health-related quality of life, healthcare resource utilization, and physical function compared with usual care^18–20^. It is estimated that 74–85% of trial assessments could be collected remotely, potentially reducing the number of physical visits by up to 40%^21^. This capability aligns with the current movement towards decentralized clinical trials that could deliver greater patient centricity by minimizing geographic or logistical barriers to participation^6^. This could not only potentially support increased trial sample diversity but also provide opportunities for outpatient care delivery instead of prolonged hospitalization.

Following a slow but steady increase in the use of decentralized and digital technologies in clinical trials between 2014 and 2019, there was a sharp rise in the usage of and reliance on RPM tools in clinical trials during the COVID-19 pandemic, owing to the need to minimize the spread of infection; however, usage has declined since the height of the pandemic^8,22^. Reversing this trend and increasing the use of RPM in clinical trials, therefore, represents an opportunity to improve efficiency, safety monitoring, inclusivity, and data quality.

RPM solutions have the potential to transform clinical trial delivery and benefit patients, HCPs, and study sponsors; however, there are obstacles that need to be overcome to integrate RPM into clinical trials effectively. This article describes key advancements in RPM technology and provides a framework for developing fit-for-purpose RPM solutions while highlighting challenges that need to be overcome to enable more digitalized, patient-centric approaches to innovation of clinical trial design and delivery.

## A framework for developing fit-for-purpose solutions

### Needs assessment, scientific strategy, and development of the conceptual model

Novel, multimodal RPM solutions that combine remote data collection via patient-completed questionnaires with self-administered medical devices must be supported by robust scientific and clinical evidence, with particular importance on the combination of suitable technology with clinical and measurement science to ensure that RPM solutions are fit for purpose^23^. The expanding use of monitoring tools in consumer wellness contexts should not preclude medical device or RPM providers from applying the highest standards of evidence generation for safe use and performance of their solutions, whether they are subject to regulatory oversight or not^23,24^.

The needs assessment should also define the anticipated value proposition for the RPM solution, including the expected balance between added implementation costs and potential efficiencies for patients, sites and sponsors^25^. Costs and efficiencies can be considered across a range of areas, including device provision, training, technical support, notification management, and integration with trial systems or clinical workflow, as well as potential offsets from fewer site visits, improved retention, earlier issue detection or more efficient data collection.

### Prototype development

**Fig. 1:**
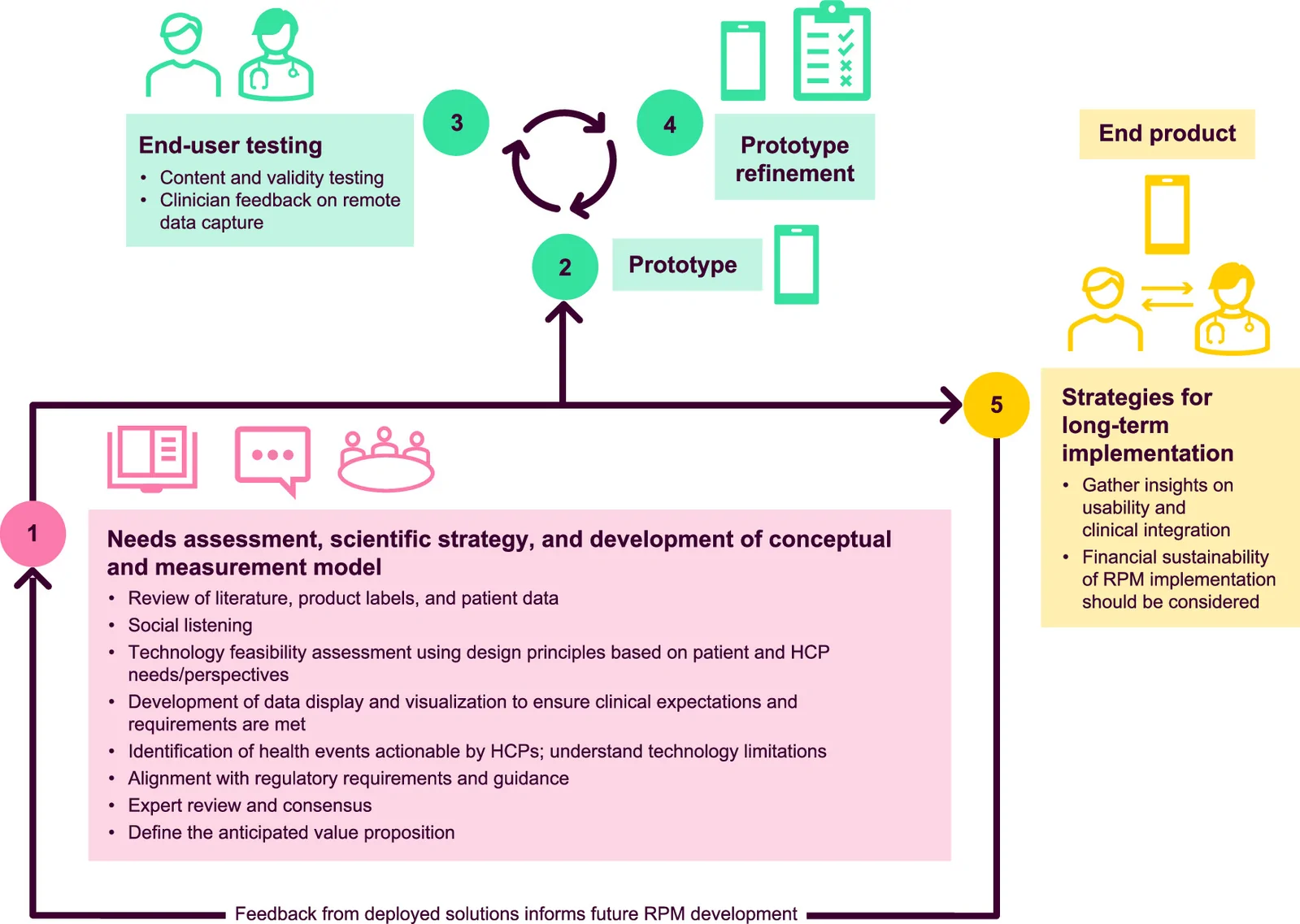
A framework for developing fit-for-purpose RPM solutions. HCP healthcare professional, RPM remote patient monitoring.

The content development and design process for an RPM solution includes several key steps (Fig. 1). The development of an RPM begins with conceptualizing the construct to be measured, such as symptoms of a toxicity that is of specific interest because of its morbidity, impact on optimal dosing and/or trial retention. Conceptualization draws on literature reviews including clinical guidelines, toxicity grading criteria, social listening, expert review and, if needed, expert consensus (e.g., when clinical manifestations predictive of a toxicity risk are too unspecific in grading criteria). Comprehensiveness of data capture is not the primary goal of such a solution, but rather the HCP perspectives on data utility and clinical relevance for monitoring and triaging patients who might require a clinical evaluation. The identified desired outcomes are mapped to the high-level scientific requirements for data acquisition, data visualization and notifications. Patient and HCP expectations should also be explored to inform the design of RPM app interfaces, including visual elements, tone, and terminology to support sustained engagement.

Design should be guided by behavioral and measurement sciences and key user experience (UX) design principles so that the RPM solution enables patients to provide reliable insights to support HCPs when monitoring patient risk of morbidity. To collect reliable patient-reported data, it is important to remove barriers to inputting data (e.g., by providing intuitive user interfaces and educational content, and by simplifying medical terminology) while providing content that is easy to access, facilitating meaningful interactions that keep patients engaged and motivated throughout their journey, and fostering trust between the patient and HCP. Easy access to information and guidance at each stage of the journey also allows patients to understand the impacts of their actions and the interventions and to take a more active role in the management of their condition, potentially improving their overall experience.

Collecting clinical measurements in the patients’ homes rather than from controlled clinical environments might introduce human-factor risks that should be accounted for in RPM solution design^26^.

### End-user testing and prototype refinement

The resulting concept prototype is then tested extensively for content and validity with intended users (i.e., patients and HCPs). Key priorities at this stage, which could differ from the development of traditional health evaluation questionnaires, include aligning with clinical expectations for signal detection, enhancing the clarity and interpretability of the data, and ensuring the platform can scale effectively across different use cases and populations. It is important to understand if there are any limitations of the technology that may pose clinical risks (e.g., over-reliance), delayed entry of data by the patient or delayed evaluation of notifications by the HCPs. Additionally, regulatory and quality compliance reviews are required for alignment with regulatory requirements and guidance for digital health solutions implemented in clinical trials^23,24^.

Prototype development, end-user testing, and prototype refinement are all parts of a development loop to refine the RPM solution for long-term deployment.

### Strategies for long-term implementation

As part of the development process, it is also vital to devise a strategy for long-term implementation of the RPM solution by gathering insights on anticipated challenges related to clinical acceptance and integration, and analyzing data from the RPM deployment to inform the scalability and transferability of the solution across other contexts.

RPM solutions must integrate with site workflows, with alerts routed to patient management staff rather than protocol monitors. HCPs are often faced with an overwhelming amount of data, notifications, and required actions regarding their patients, together with expanding regulatory requirements. RPM solutions should therefore aim to provide relevant data and behavioral nudges in a timely and focused manner and to reduce the administrative burden, allowing HCPs to concentrate on the most important issues and tasks. Patient-reported data need to be transformed into clear, actionable, visual formats that support HCPs and wider clinical trial teams in making collaborative, timely, and informed decisions.

By applying a robust approach, in the context of clinical trials, RPM should enable near real-time data collection to help HCPs focus on the most important information and develop a more comprehensive understanding of patients’ disease states and symptom profiles, possible worsening in signs/symptoms, adherence to medication schedules or assessments, and/or treatment toxicity. In the case of symptom assessment, trials have demonstrated that the use of RPM for systematic self-reporting of patients’ symptoms enabled earlier and more frequent reporting than during routine visits, allowing timely intervention and mitigating HCP underestimation of patient symptoms^18,20^. Implementation of electronic symptom monitoring is also possible in multicentre trials, supporting the scalability of RPM for toxicity surveillance in large trials^27^. In addition, RPM has the potential to improve medication adherence in trials. Poor medication adherence can limit the analysis and interpretation of outcomes in clinical trials; typical methods for assessing adherence (e.g., capsule counts and periodic interviews/questionnaires) are of limited effectiveness^28^. However, the use of mobile app-based interventions has been shown to improve medication adherence^29^, and similar strategies could promote participant adherence to actions required for outcome assessments.

Financial sustainability should be considered as RPM solutions may introduce upfront or recurring costs related to their implementation, for example, the need to develop technology platforms, provide connected devices, and to arrange training and support. However, financial planning for digital health interventions should be considered in the context of broader sources of value that may not be captured in conventional evaluations of drugs or devices, including reduced participant travel and fewer avoidable visits, improved retention and more efficient trial conduct^25^. A modeling study of decentralized clinical trials has estimated that applying decentralized methods in phase II and III trials could generate substantial financial value for sponsors^30^.

### Examples of fit-for-purpose RPM solutions in clinical trial delivery

Recent deployments of fit-for-purpose RPM solutions in oncology clinical trials have demonstrated the potential to enhance patient care and trial delivery. This section provides examples of digital tools created to enable the monitoring of skin toxicities and stomatitis, which were developed and tested among small cohorts of patients and HCPs before being deployed in clinical trials^31,32^. To support the self-management of stomatitis in patients undergoing systemic cancer therapy, an RPM platform was co-designed with patients, nurses, and safety scientists^32^. The development was informed by behavioral and measurement science principles, with features including symptom tracking, tailored self-management advice, and secure data sharing. The RPM solution included a patient-facing mobile phone app, which provided self-management tips to encourage adherence to prophylactic treatment, with illustrations to visually cue adherence to the oral care protocol of the trial. The app also prompted patients to contact the clinical site team when symptoms reached a given morbidity threshold, with HCPs receiving relevant and timely insights into patient status via a dedicated digital portal that enabled them to visualize data on demand, thereby helping to address the gaps in patient monitoring between clinic visits. Upon worsening patient status, HCPs were prompted to review patient data by digital notifications. Preliminary feedback from patients noted a low burden of daily usage and suggested that the solution was beneficial for monitoring symptoms and facilitating communication with clinical teams. Findings indicated that the RPM platform enables earlier intervention and augments existing resources for stomatitis management.

For remote monitoring of skin toxicities in patients receiving cancer treatment, a digital RPM solution was designed following a similar approach to the above example for stomatitis, incorporating user-centric principles, with validation involving both patients and HCPs^31^. Similar to the solution for stomatitis monitoring, HCPs were provided with a web portal to receive updates on patient status, and patients interacted through a smartphone app that displayed an interactive body avatar that allowed patients to annotate symptom locations and to upload photographs for examination by HCPs. Content validation and qualitative user testing demonstrated that the RPM solution reliably captured symptom data and was well received by users, who found it helpful for tracking symptoms and accessing educational material. HCPs valued the improved insight into patient status between visits, and the RPM solution was noted to facilitate earlier identification and management of skin toxicities. Further research is planned after implementation of the RPM solution in clinical trials to establish its clinical utility and validity in larger cohorts from clinical trials.

### Overcoming challenges to drive adoption of RPM in clinical trials

To deploy RPM strategies in clinical trials, it is important to understand the potential barriers, including regulatory uncertainty, data privacy, and variability in digital literacy, so that strategies can be developed to overcome these issues. Regulatory agencies have published guidance on the use of RPM in decentralized trials, including the European Medicines Agency’s recommendation paper on decentralized elements in clinical trials^33^ and the US Food and Drug Administration’s guidance on digital health technology for industry, investigators, and other stakeholders^23^, and it is important for trial sponsors and site staff to be aware of and follow such guidance. Multiregional clinical trials can be particularly challenging given inter-regional variations in regulations^8^. The potential role of a clear, fit-for-purpose, risk-based regulation pathway in addressing this issue was recently highlighted^8^.

Regarding data privacy and security, RPM involves the transmission of personal data and, as such, must be fully compliant with local regulations (e.g., the US Health Insurance Portability and Accountability Act and the General Data Protection Regulation of the European Union). Regulatory recommendations from the US Food and Drug Administration and the European Medicines Agency provide advice on how to mitigate privacy-related risks^23,33^. Consideration of alignment with regulatory requirements and all relevant guidance and regulations during development, as specified in the framework detailed in this article, helps to address these regulatory and privacy concerns.

A potential disparity could arise in clinical trial participation between participants of different demographics and socioeconomic backgrounds owing to variations in digital literacy, which also varies among healthcare staff^34^. To increase inclusivity, training and technical support should be provided, and RPM solutions should be designed with user-friendly interfaces and accessibility in mind. Considering patient needs and soliciting patient input on RPM app interfaces, visuals, tone, and terminology as part of the RPM development framework serve to increase inclusivity. Disparities in patient technology access were ranked by investigators as one of the top three disadvantages of using RPM in clinical trials^22^, despite the fact that mobile phone and internet access are now thought to be widespread^35,36^. Technology access and digital literacy may be a barrier for a minority of potential participants; therefore, the provision of devices may sometimes be necessary to maximize inclusivity. This may also be necessary if apps and connected devices for a given RPM solution have more advanced system requirements than participants’ devices. However, provisioning devices likely increases costs for the trial sponsor, and the bring-your-own-device approach is preferred by approximately half of patients^37^. Any new technology or process should be integrated as seamlessly as possible into the existing workflows of HCPs, who are likely dealing with high workloads, and developers should avoid creating ‘alert fatigue’ through excessive notifications^38^. Consideration of these challenges for HCPs and the incorporation of HCP input throughout the design and development process, as detailed in the aforementioned framework, facilitates optimal integration and workflow.

A previous lack of evidence and uncertainty regarding overall improvements in efficiency and costs in clinical trials adopting RPM tools may also have contributed to the recent decline in the use of RPM^8^. The emergence of additional RPM solutions developed within a framework designed to ensure feasibility and practicality may provide the robust scientific evidence required and result in future increases in the use of these tools and the realization of their promise in reducing the burden and improving the efficiency of clinical trials.

### Conclusions

Although RPM technologies have been commonplace in certain aspects of routine clinical care for some time, the use of RPM in clinical trials was galvanized by the need for remote trial participation during the COVID-19 pandemic. RPM solutions for use in a clinical trial setting must be designed and developed within carefully considered frameworks. They must be supported by robust evidence, while overcoming key barriers to their successful deployment, namely uncertainty about regulations and data protection among trial sponsors and staff, and factors affecting trial participation and inclusivity. Successful deployment of RPM solutions in clinical trials will require not only regulatory clarity, but also rigorous attention to human-factor risks when patients collect clinically relevant data unsupervised. Following the framework described herein, there is the potential for RPM solutions to provide opportunities in clinical trial settings to improve aspects of data collection and sharing with HCPs and may enable more efficient clinical trial delivery. Evidence suggests that RPM may improve patient engagement in trials, with more accurate symptom reporting than during routine visits and better adherence to medication schedules^18,20,29^.

## Data Availability

Data sharing is not applicable to this article because no datasets were generated or analysed during the current study.
